# Retrograde intramedullary headless compression screw fixation for pediatric mid-diaphyseal proximal phalanx malunion: A case study

**DOI:** 10.1016/j.ijscr.2025.110824

**Published:** 2025-01-04

**Authors:** Ismaël Maes, Arne Decramer, Bert Vanmierlo

**Affiliations:** a Faculty of Medicine, UZ Leuven, Herestraat 49, 3000 Leuven, Belgium; b Department of Orthopaedics and Traumatology, AZ Delta, Deltalaan 1, 8800 Roeselare, Belgium; c KULeuven-Leuven University, Department of Development and Regeneration, Herestraat 49, 3000 Leuven, Belgium; d Department of Cardio and Organ Systems, Hasselt University, Martelarenlaan 42, 3500 Hasselt, Belgium

**Keywords:** Fracture, Intramedullary, Malunion, Phalanx, Screw

## Abstract

**Introduction:**

Proximal phalanx fractures in children, especially mid-diaphyseal fractures, can result in malunion and significant functional impairment. Early malunions require prompt and effective intervention to prevent long-term complications. This case study highlights the use of intramedullary headless compression screw (IMHCS) fixation in addressing a proximal phalanx malunion.

**Case presentation:**

A 12-year-old boy presented with a malunion of the mid-diaphyseal proximal phalanx of the fourth finger following conservative treatment of a cycling injury. Initial management involved immobilization followed by buddy taping; however, incomplete radiographic evaluation resulted in an underestimation of the volar angulation. At the four-week follow-up, the patient exhibited 50° volar angulation, clinodactyly, and marked stiffness. The malunion was treated surgically with retrograde IMHCS fixation after osteoclasis. Radiographic evaluation confirmed proper reduction and alignment. The patient began physical therapy immediately, achieved full range of motion within four weeks and maintained excellent functional outcomes at one year postoperatively.

**Discussion:**

Retrograde IMHCS fixation is an innovative technique for managing phalangeal malunions, providing stable fixation and enabling early mobilization. This method avoids the physis, minimizing the risk of growth disturbances, eliminates the need for hardware removal, and ensures proper alignment.

**Conclusion:**

IMHCS fixation is a promising solution for early malunions and potentially fresh fractures of the proximal phalanx in pediatric patients. It offers stable fixation, preserves physeal integrity, and supports early rehabilitation, contributing to excellent functional recovery. Further studies are needed to evaluate its long-term outcomes.

## Highlights

• Complexity in Pediatric Fractures: Managing pediatric proximal phalanx fractures, especially mid-diaphyseal, poses challenges due to soft tissue forces and potential growth disturbances.
• Limitations of Traditional Methods: Conventional treatments like K-wire fixation risk physeal damage, delayed recovery, and suboptimal outcomes.
• IMHCS as an Optimal Solution: The intramedullary headless compression screw provides stability, preserves the physis, allows immediate physiotherapy, and avoids hardware removal.
• Successful Application and Outcome: A 12-year-old patient treated with retrograde IMHCS showed full motion recovery and no growth issues.
• Future Recommendation: IMHCS fixation is minimally invasive and suitable for both malunions and fresh fractures in pediatric patients.

## 1 Introduction

Phalangeal fractures are common hand injuries, with fractures of the proximal phalanx comprising 38.7 % of all phalangeal fractures [1]. These fractures can be categorized into three primary types: those involving the phalangeal base, the shaft (diaphysis), and juxta-epiphyseal regions. Diaphyseal fractures of the proximal phalanx are less common than fractures at the proximal or distal ends. They often result from sports-related injuries in older children and adolescents, and typically result from an extension-type trauma and exhibit apex volar angulation. The interosseous muscles attach onto the base of the proximal phalanx and flex the proximal fracture fragment, while the flexor and extensor tendons impart a longitudinal compression force that shortens the phalanx and extends the distal fragment. This interplay of soft tissue structures complicates the management and treatment of these fractures. While the majority of proximal phalangeal fractures can be effectively treated nonoperatively, surgical intervention is warranted in certain cases. Surgery should be considered for fractures that are length-unstable (such as spiral, oblique, or comminuted shaft fractures), those presenting with rotational deformity, fractures with more than 25° of dorsal or palmar angulation, or over 10° of radial or ulnar angulation following an attempted reduction. Most fractures involving the phalangeal base or shaft that require surgical management can be addressed using closed reduction and percutaneous pinning [2]. In cases of fresh malunion of the proximal phalanx in pediatric patients, particularly those with rotational deformity and significant stiffness, intramedullary headless compression screw (IMHCS) fixation presents a compelling alternative to conventional pinning. This technique provides stable fixation, minimizes soft tissue disruption, and facilitates immediate, intensive physical therapy, thereby enhancing the potential for improved functional outcomes.

## 2 Case report

**Fig. 1:**
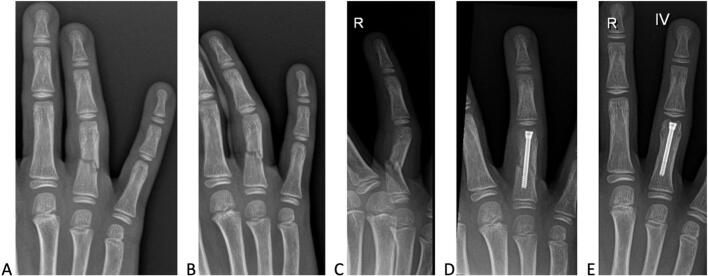
A,B Classical postero-anterior view and ¾ view of the right hand at presentation of the patient at the emergency service. C Malunion of the fracture, showing increased displacement, shortening and angulation. D Postero-anterior view of the ring finger, two days postoperatively demonstrating good alignment, reduced angulation, callus formation and preserved joint relationships. E Postero-anterior view of the ring finger, one year postoperatively indicating stable position, no signs of material fracture or loosening, normal growth cartilage discs, and complete consolidation.

In this case report, prepared in adherence to the SCARE 2023 guidelines for surgical case reporting [3], we present the treatment of a twelve-year-old Caucasian male with a mid-diaphyseal fracture malunion of the proximal phalanx of the right ring finger (Fig. 1). The patient consulted his general practitioner with pain and swelling in the finger following a cycling accident. Radiographic analysis included posteroanterior (PA) and ¾ views, but a strict profile view of the affected finger was missing (Fig. 1A,B). This omission led to the initial underestimation of the fracture displacement, as the volar angulation could not be properly evaluated. The attending general practitioner opted for a non-operative treatment, consisting of immobilization with a Zimmer finger splint (Zimmer Biomet, Warsaw, Indiana, US) for two weeks, followed by buddy taping the ring finger to the middle finger for an additional two weeks. The initial non-surgical treatment lasted four weeks. At the first follow-up appointment in the outpatient clinic, requested by the general practitioner, proper radiographic analysis, including a strict profile view, revealed a volar angulation of 50° (Fig. 1C). The patient exhibited no active mobility in the interphalangeal joints, and passive motion was extremely painful. Additionally, significant clinodactyly was observed during an attempt to flex the fingers. By this time, a fibrous callus had already formed. Due to the challenges of achieving stable fixation with conventional techniques and the need to initiate rehabilitation immediately [4], fracture reduction and IMHCS fixation was the preferred treatment.

Surgery was performed under general anesthesia. The procedure began with an attempt at closed reduction of the fracture. A 1.0 mm K-wire was inserted percutaneously mid-laterally and slightly dorsal to the neurovascular structures to release the fibrous callus and obtain proper fracture reduction (i.e., osteoclasis). At this stage, a longitudinal step incision was made over the central slip of the extensor apparatus. A 0.8 mm K-wire was inserted in a retrograde fashion, entering the joint space centrally through the central slip. In a single attempt, the K-wire was advanced proximally, bridging the fracture site, without violating the proximal articular surface of the phalanx. Fluoroscopic control confirmed appropriate fracture reduction and correct positioning of the K-wire. A retrograde screw insertion was subsequently performed. A Cannulated Compression Headless Screw (CCHS, Depuy Synthes, Warsaw, Indiana, US) with a diameter of 2 mm was selected. The distal articular surface of the proximal phalanx was overdrilled using the appropriate cannulated drill, with the proximal interphalangeal (PIP) joint in maximal flexion to protect the proximal articular surface of the middle phalanx. Screw position and fracture reduction were verified fluoroscopically. Finally, ring anesthesia was administered using ropivacaine.

**Fig. 2:**
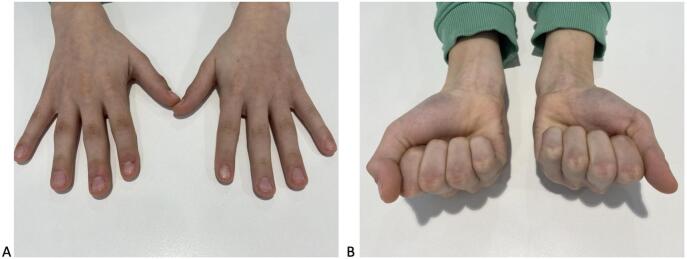
A,B One year postoperatively, the patient demonstrated full range of motion in all long fingers and symmetrical grip strength.

Two days after surgery, the patient visited the outpatient clinic. Radiographic evaluation confirmed appropriate screw positioning, satisfactory reduction, and alignment correction. (Fig. 1D). A protective orthosis and buddy loop were applied, and physical therapy was initiated without delay. The postoperative recovery was uneventful. At the four-week postoperative consultation, the patient demonstrated full restoration of finger range of motion. At the one-year follow-up, radiographs confirmed stable screw positioning without any signs of breakage or loosening, indicating complete fracture consolidation. The patient exhibited normal finger length growth and no evidence of articular degeneration at the PIP joint (Fig. 1E). The patient reported no pain, with a full range of motion in all fingers and a symmetrical power grip (Fig. 2). Removal of the hardware is neither planned nor necessary, as the screw does not interfere with the physis.

## 3 Discussion

IMHCS fixation represents a pioneering advancement in managing extra-articular proximal phalangeal fractures. The idea was suggested by Weiss, who emphasized its transformative impact on phalangeal malunions, noting minimal complications and excellent functional outcomes [5]. In the management of extra-articular proximal phalangeal fractures, as well as early malunions, like in our case, preserving the integrity of the physis is crucial. Following the same rationale, d'Oliveira and Craviotto reported using antegrade IMHCS fixation for extra-articular metacarpal fractures in an eight-year-old patient [6]. This technique effectively avoided the distally located metacarpal physis by introducing the osteosynthesis material in an antegrade manner.

When evaluating the direction of screw insertion in the proximal phalanx, Del Pinal et al. were the first to report outcomes using the retrograde technique for treating extra-articular fractures of the proximal phalanx with IMHCS fixation [7]. Their results demonstrated satisfactory outcomes with rare complications.

In pediatric patients, the retrograde approach offers notable advantages over the antegrade method. A primary benefit is the ease of placing the K-wire directly at the fracture or malunion site. Additionally, this approach avoids involvement of the proximal phalanx physis, thus reducing the risk of growth disturbances.

In a cadaveric study, Raush et al. tested torsional strength, as well as bending and distraction, in transverse distal epiphyseal fractures of the proximal phalanx. Stabilization methods included crossed K-wires, plate and screw osteosynthesis, and retrograde IMHCS fixation with a partially threaded screw. The findings indicated no significant difference in load to failure between IMHCS fixation and plate-and-screw fixation [8].

Similarly, Miles et al. compared plate-and-screw osteosynthesis with IMHCS fixation in a fixed fracture model of the proximal phalanx. Their study revealed that both techniques provided equivalent stability after cyclic loading [9]. These findings further highlight the reliability of IMHCS fixation as an effective alternative to traditional stabilization methods.

Managing proximal phalangeal malunions in children requires a nuanced approach to restore function and correct deformities. The treatment strategy often depends on factors such as the child's age, the degree and direction of the deformity, the time elapsed since the injury, and, importantly, the level of the malunion. Due to the highly active periosteum in children, substantial callus formation can occur within 2–3 weeks, often resulting in a lack of tenderness at the fracture site during examination. In these cases, surgical intervention is frequently indicated. The surgeon should be prepared to use fluoroscopic guidance and a K-wire to percutaneously manipulate the fracture and disrupt the developing callus (osteoclasis) to correct the alignment [10].

Although early-stage malunions are relatively common in pediatric phalangeal fractures, there is a notable scarcity of literature on the subject. A review by Al-Qattan et al. explored the natural remodeling capacity of the proximal phalanx in neck fractures [11]. While significant remodeling often occurs in malunions within the sagittal plane, this process is less effective in cases involving the lateral plane or rotational deformities, where surgical intervention is typically required. A case series by Waters et al. focused on the management of proximal phalangeal neck malunions in children, describing the use of osteoclasis with a K-wire, which acted as a lever arm to facilitate fracture reduction. Following reduction, a separate K-wire was inserted percutaneously to stabilize the fracture. This technique effectively addressed bony obstructions to flexion, resulting in an average improvement of 41.7° in flexion among the treated patients. For fresh diaphyseal malunions, a similar approach to acute fracture management can be applied, with K-wire fixation providing reliable stabilization of the phalanx following reduction. However, in both manuscripts, no specific conclusions are drawn regarding the management of phalangeal shaft malunions.

Although K-wire fixation is minimally invasive and known for its swift procedural time, maximal elastic force is typically low. This necessitates prolonged immobilization and delays functional rehabilitation [4]. Furthermore, crossed K-wire fixation often penetrates the physis, potentially disturbing growth due to iatrogenic epiphysiodesis. The application of the technique of the retrograde IMHCS fixation following osteotomy in proximal phalanx fracture malunions, which is comparable to our case, was evaluated through in vitro biomechanical analysis by Deschuyffeleer et al. In their cadaveric study, mean maximal force and stiffness were assessed using a three-point bending test. The results demonstrated stability comparable to that achieved with plate and screw osteosynthesis [12].

A clinical study by Caekebeke et al. further validated the technique, reporting excellent outcomes in four adult patients with satisfactory early postoperative results [13]. Similarly, Weiss described the use of retrograde screw insertion for phalangeal malunion in his manuscript [5]. However, the focus of this work was primarily on the technical aspects of the procedure, without specifying the number of patients treated, their age, or providing detailed insights into the approach to addressing the malunion. This leaves certain aspects of its clinical application unclear.

However, this technique has certain drawbacks. One significant limitation is that the rotational stability observed in transverse epiphyseal fractures of the proximal phalanx, as reported in the study by Raush et al., cannot be directly extrapolated to the management of transverse shaft fractures [8]. Further research is essential to assess rotational stability specifically in phalangeal shaft fractures.

Another notable drawback is the relatively smaller distal articular surface compared to the proximal one, which might increase the risk of iatrogenic degenerative arthritis from screw insertion through the PIP joint. Nevertheless, a large systematic review by Hug et al., encompassing 837 patients treated with IMHCS for metacarpal and phalangeal fractures, found no evidence of postoperative articular degeneration [14].

In our patient, the phalangeal fracture was not fresh, but rather a malunion in progress, which already significantly impacted mobility and function. The ability to immediately start physiotherapy after reducing the malunion and stabilizing the fragments is crucial to ensure a good outcome, in such traumatic cases. This technique is effective in adults and has the added advantage in pediatric patients of not affecting the physis during fracture fixation. Additionally, the biomechanical impact of screw insertion on joint cartilage, underscores the need for careful surgical technique and selection of appropriate screw dimensions to minimize iatrogenic damage.

In conclusion, the retrograde IMHCS provides excellent fixation after reduction of an early malunion of the proximal phalanx in a pediatric patient. The technique could also be effective in fresh fractures. The construction is exercise stable and the physis is avoided during surgery, minimizing disruption of the physis.

## CRediT authorship contribution statement

The idea and content of the manuscript were conceived by BV. All clinical assessments and the surgical procedure were performed by BV. Data collection and interpretation, including radiographic analysis, were conducted by IM and BV. The initial draft of the manuscript was written by IM and BV. BV, IM and AD reviewed, edited, and approved the final version of the manuscript.

## Informed consent

Written informed consent was obtained from the patient's parents/legal guardian for publication of this case report and any accompanying images. A copy of the written consent is available for review by the Editor-in-Chief of this journal on request.

## Guarantor

Dr. Bert Vanmierlo accepts full responsibility for the work and conduct of this study. He had access to the data and controlled the decision to publish the findings.

## Funding statement

The authors received no financial support for the research, authorship, and/or publication of this article.

## Registration of research studies

1. Name of the registry: OSF registries

2. Unique identifying number or registration ID: B1172024000017

3. Hyperlink to your specific registration (must be publicly accessible and will be checked): doi:10.17605/OSF.IO/X2K6A

## Ethical approval details

Ethical approval to report this case was obtained from the ethical board (Study number 24093; BUN B1172024000017).

## Declaration of competing interest

The authors declare no potential conflicts of interest with respect to the research, authorship, and/or publication of this article.
